# Influence of social networks on cancer survivors' self‐management support: A mixed methods study

**DOI:** 10.1111/ecc.13578

**Published:** 2022-04-13

**Authors:** Gilly Howard‐Jones, Ivaylo Vassilev, Debora Fenlon, Sean Ewings, Alison Richardson

**Affiliations:** ^1^ Department of Health Sciences University of Southampton Southampton UK; ^2^ Maggie's Cancer Support Centre Southampton University Hospital Southampton Southampton UK; ^3^ Department of Medical Statistics University of Southampton Southampton UK; ^4^ University of Southampton & University Hospital Southampton NHS Foundation Trust Southampton UK

**Keywords:** cancer survivors, mixed methods, self‐management, social networks, social support

## Abstract

**Objective:**

The role of social networks, especially weaker ties (e.g. casual acquaintances and hobby groups), in self‐management of long‐term consequences of cancer is unexplored. This study aimed to explore the structure of cancer survivors' social networks and their contribution to self‐management support and health‐related quality of life (HRQoL).

**Methods:**

The study used a sequential, exploratory mixed methods design. Phase 1 surveyed 349 lymphoma, colorectal, breast and prostate cancer survivors. Phase 2 analysed 20 semi‐structured interviews with respondents recruited from Phase 1.

**Results:**

Phase 1 results suggested participants' HRQoL increased if they participated in an exercise group, if their self‐management skills increased, and social distress and negative illness perception decreased (*p* < 0.0005 adj. *R* ^*2*^ = 0.631). These findings were explored in Phase 2, identifying underlying mechanisms. Four themes were identified: disrupted networks after cancer treatment; navigating formal support and building individual capacity; peer networks and self‐management knowledge and linking networks to enable adaptation in recovery.

**Conclusions:**

This study suggests engagement with community groups, particularly those not directly related to illness management and social interaction with weak ties, make a valuable contribution to self‐management support, increase HRQoL and enhance well‐being.

## 1 INTRODUCTION

The role that social networks and connections play in shaping a person's behaviour and subsequent impact on health and well‐being is increasingly recognised. Seminal studies demonstrated relationships between increased social engagement and reductions in mortality and morbidity (Berkman & Syme, 1979; Christakis & Fowler, 2007; House et al., 1988). The influence of social networks on self‐management support for long‐term conditions, such as diabetes, suggests that having a diverse network increases social connectedness and satisfaction with current networks and associated with enhanced self‐management skills, physical and mental well‐being (Vassilev et al., 2016). This may in part be due to diverse social networks providing greater access to informal practical resources (Kroenke et al., 2013). However, larger social networks and networks including different types of relationships can also require higher levels of relationship management (Vassilev et al., 2019) and be emotionally burdensome to manage. This indicates that the underlying mechanisms through which social networks operate are complex and that networks may have negative, as well as positive, impacts on health and quality of life (Cheng et al., 2013; Hamilton et al., 2010; Vassilev et al., 2016).

Previous cancer social network studies, largely undertaken in women with breast cancer, have found associations between increased network size, overall survival and cancer survival (Beasley et al., 2010; Jones & Storksdieck, 2019; Kroenke et al., 2006, 2017; Lindstrom & Rosvall, 2019; Sarma et al., 2018; Waxler‐Morrison et al., 1991). Other studies have identified relationships between higher levels of social network engagement and higher HRQoL (Cheng et al., 2013; Kroenke et al., 2013; Lim & Zebrack, 2006; Soares et al., 2013), lower inflammatory markers and depressive symptoms (Hughes et al., 2014), increased exercise engagement (Kim et al., 2015) and increased support for healthy eating (Crookes et al., 2016). Social support has been found to be valuable in self‐management, but there is limited research exploring network characteristics or utilising social network approaches and theories (Balfe et al., 2017; Henshall et al., 2018; Kim et al., 2020; Paterson et al., 2015). Few studies have examined how Socio‐Economic Status (SES) could influence social network access to self‐management resources for people with cancer or long‐term conditions (Juárez‐Ramírez et al., 2015; Reeves et al., 2014).

As the incidence of those living with and beyond cancer is predicted to rise (Maddams et al., 2012), self‐management support has been adopted as an approach to meet increasing health and well‐being needs of cancer survivors (Batehup et al., 2017). Self‐management has limitations as it frequently focuses on individual concerns, such as relapse (Fenlon et al., 2015) and does not consider how personal agency, shaped by social networks can influence self‐management behaviour outcomes (Dunn et al., 2021).

**FIGURE 1:**
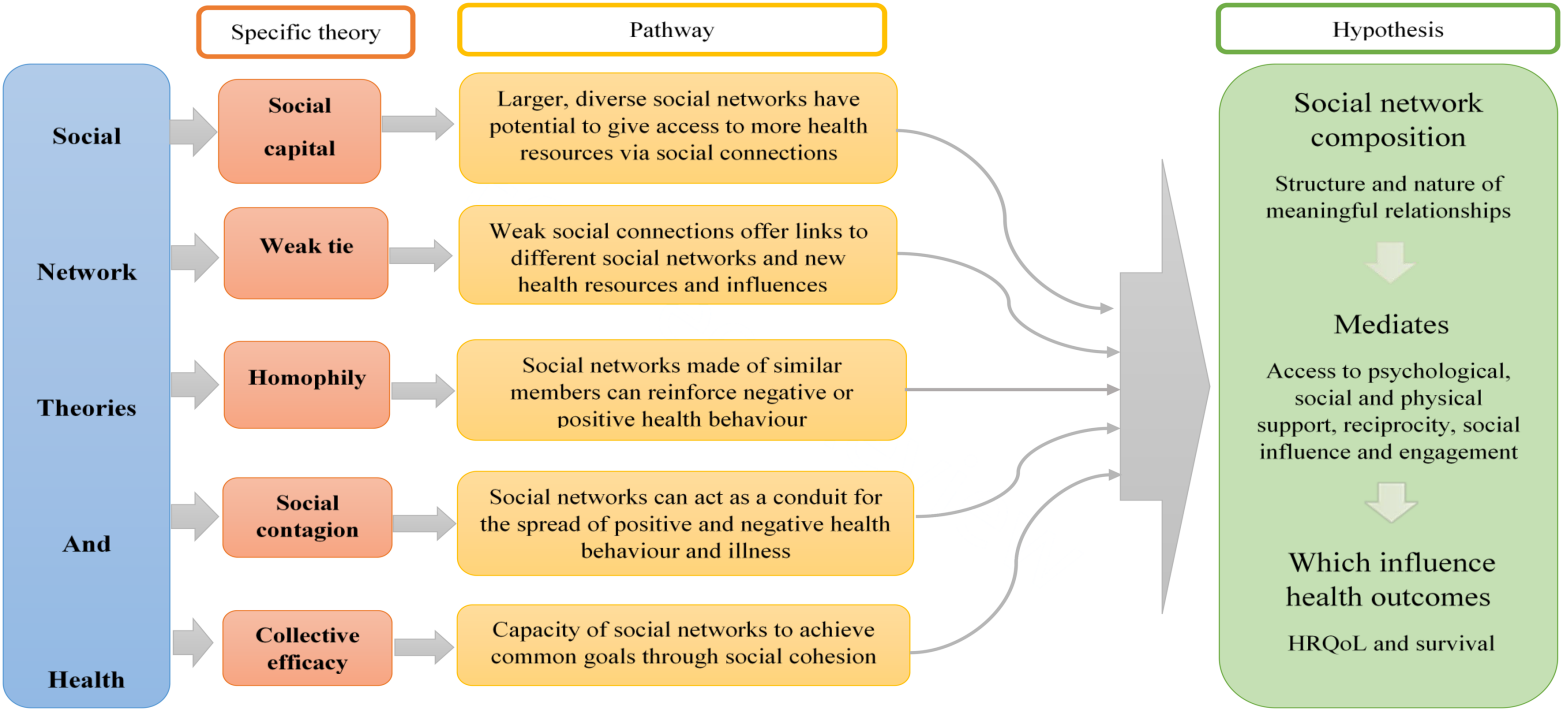
Social network theories and application to health framework

Drawing on social network theories (Figure 1), the aims of this study were to contribute towards the development of a contextualised understanding of self‐management and self‐management support by describing the dimensions of personal social networks (characteristics and social engagement) of cancer survivors; determining if social network dimensions and social distress are associated with health‐related quality of life, exploring how social networks contribute to patients' efforts to self‐manage survivorship concerns and determining if social network characteristics are associated with SES.

## 2 METHODS

### 2.1 Study design

A mixed methods design, utilising an explanatory sequential approach was chosen to address study aims and reported using GRAMMS guidelines (O'Cathain et al., 2008). Data from Phase 1, a quantitative cross‐sectional survey and Phase 2, qualitative semi‐structured interviews, were collected concurrently and reported using SRQR guidelines (O'Brien et al., 2014). Survey was the dominant method, its purpose to quantitatively characterise social network dimensions of cancer survivors and contribution to self‐management. Phase 1 results informed analysis of Phase 2, enabling description and analysis of social network mechanisms. Ethical approval was gained from East of England Ethics Committee‐Essex on 13 May 2015 reference number 15/EE/0137.

### 2.2 Participants and procedures

Participants were recruited from five NHS Trusts in England between August 2015 and June 2016, while attending hospital appointments or approached by post, following identification through hospital databases. Participants completing Phase 1 were invited to participate in Phase 2.

Respondents were 18 years or older, diagnosed and received primary treatment for breast, bowel, prostate cancer, non‐Hodgkin's or Hodgkin's lymphoma and remained disease free (maximum 24 months since diagnosis). Research nurses identified and recruited survey participants. Principal investigator (G. H. J.) received survey responses and recruited interview participants.

### 2.3 Phase 1: Survey

**FIGURE 2:**
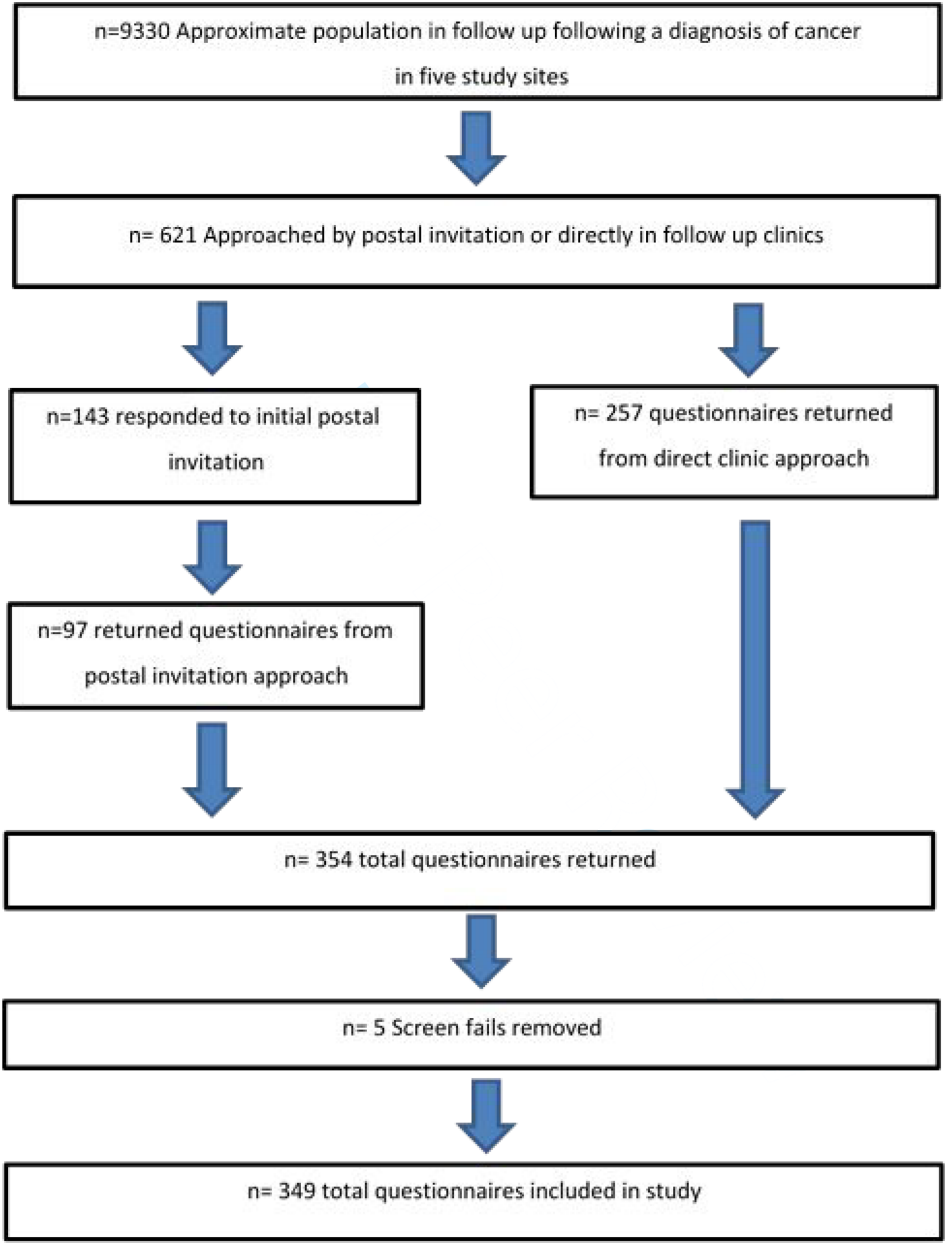
Flow chart of recruitment of participants in the study

Over 11 months 621 participants meeting the eligibility criteria were approached across 5 Trusts, with 349 consenting to Phase 1 of the study, eliciting a 56% response rate (Burns & Grove, 2003) (Figure 2). The survey consisted of five components:

### 2.4 Health related quality of life

HRQoL data were collected using the Functional Assessment of Cancer Therapy‐General (FACT‐G) (Cella et al., 1993) because it is generalisable to all people with a cancer diagnosis.

### 2.5 Participant social engagement and characteristics

Demographic data were collected on cancer diagnosis and treatment. SES was determined using the Indices of Multiple Deprivation, derived from participants' postcode (Gov.uk, 2015). Engagement in social activity data, for example, recreation, were collected using a section from a questionnaire developed by Vassilev et al. (2013) and previously used with participants with long‐term conditions. Face validity was gained through Patient and Public Involvement (PPI) review and pilot study (Table 1).

**TABLE 1:** Phase 1 participant characteristics

Characteristics	Total number	Breast number	Prostate number	Lymphoma number	Colorectal number
Age					
Years (mean)	62.91	57.9	70.7	59.2	68.3
Gender					
Female	195	138	0	39	19
Male	151	0	80	36	35
Missing	3				
Ethnicity					
White	341	134	81	72	54
Marital status					
Married/civil partner	250	96	63	51	40
Divorced or widowed	74	23	14	15	10
Never married	26	13	3	7	3
Education attained					
No qualifications	53	21	13	11	8
School cert/GCE/O level	217	89	46	54	28
A levels	102	40	25	21	16
Vocational	255	99	59	49	46
Degree	110	45	22	26	17
Working status					
Employment	134	69	12	29	22
Long‐term sickness	14	6	1	4	1
Looking after home/family	24	11	1	6	4
Voluntary work	24	7	11	3	1
Education	4	0	0	2	0
Retired	183	49	66	35	30
None of above	12	5	1	4	0
Tenure					
Owns/mortgage	300	118	71	65	49
Rents	45	20	9	9	4

### 2.6 Social network characteristics

A social network assessment tool was developed based on the name generator approach (Vassilev et al., 2013). Participants identified all members of their social network by name, describing their relationship to them (e.g. ‘Sarah’ and ‘work friend’). Participants scored each member's contribution to their self‐management support (0 = *never* to 5 = *a lot*) in three domains: illness work (e.g. managing medication), day to day work (e.g. housework) and emotional work (e.g. someone to talk to about worries) and indicated how close members lived to them, for example, a short walk. Face validity was achieved through PPI review and pilot study.

### 2.7 Self‐management

Data were collected using subsection 4 ‘self‐monitoring and insight’ and 6 ‘skill and technique acquisition’ from the Health Education Impact Questionnaire (HEIQ) (Osborne et al., 2007), previously validated in cancer populations (Maunsell et al., 2014).

### 2.8 Social distress

Data were collected using the Brief Illness Perception Questionnaire (BPIQ) (Broadbent et al., 2006), adapted with permission for use in a cancer population. Face validity was gained through feedback from PPI group and pilot study. Cronbach's alpha was undertaken to test internal reliability. The result was 0.68, under the recommended 0.7, but lower results are acceptable within psychological constructs (Klein, 1999). Social Distress data were collected using the Social Difficulties Inventory (SDI) (Wright et al., 2011).

### 2.9 Statistical analyses

Descriptive statistics are used to summarise demographic and social network dimensions of the sample. Preliminary analyses were conducted to ensure there was no violation of assumptions of normality, linearity, homoscedasticity and multicollinearity. Preliminary linear regression was undertaken, followed by four multiple regression models which observed for relationships between HRQoL and participant characteristics, social network characteristics, network contribution to self‐management and social distress. A final multiple regression model was undertaken with all previously significant variables. Statistical analysis was undertaken with SPSS® software, version 25.

### 2.10 Phase 2: Semi‐structured interviews

A purposeful sample of 20 participants reflecting age, gender, disease and SES was selected for Phase 2. Interviews were recorded with consent and undertaken at participants' choice of location. The interview guide was developed from work by Reeves et al. (2014), collecting narrative data on participants' experiences on the role of social networks in self‐management support. Sample size was considered large enough to offer meaningful analysis (Kvale, 2007; Quin Patton, 2002; Tashakkori & Teddlie, 2010). Techniques to enhance trustworthiness of data collection were addressed by (R. A.) and (I. V.) critically reviewing a sample of interview recordings (Lincoln & Guba, 1985). The dynamic relationship between the interviewer and interviewees was acknowledged. The interviewer kept a critical journal to reflect on each interview and minimise researcher influence (Bryman, 2016; Spradley, 1979).

### 2.11 Data analyses and interpretation

Interview findings were analysed deductively, using framework analysis (Gale et al., 2013). Themes identified from survey findings and informed by weak tie theory (Granovetter, 1973) were used to describe and explain how weak tie (non‐familial and peripheral) social network members, social network mechanisms and SES influenced self‐management support. Data were imported into the software programme NVivo 12 to facilitate analysis (Ritchie & Lewis, 2014). G. H. J. conducted the analysis; subsamples of which were independently coded by A. R. and I. V.

### 2.12 Data integration

Integration occurred throughout the study. The same sample of participants generated quantitative and qualitative data to explore the subject from different perspectives. Survey findings informed analysis of interview data to gain further understanding of the mechanisms that could explain observed statistical trends. Data from Phases 1 and 2 were used to address the aims of the study and key findings were used to derive a multi‐faceted knowledge of the influence of social networks on cancer survivors' self‐management support (Bazeley, 2018; O'Cathain et al., 2007; Teddlie & Tashakkori, 2009).

## 3 RESULTS

### 3.1 Phase 1

A total of 349 people were recruited, 56% women (*n* = 195) and 44% men (*n* = 154). Mean age was 63 years (standard deviation 13.04). Women with breast cancer were the largest diagnostic group 39% (*n* = 138), followed by men with prostate cancer 23% (*n* = 82), lymphoma 21% (*n* = 75) and bowel cancer 15% (*n* = 54). Participants were most frequently within 2–6 months of completing cancer treatment (38%; *n* = 131). The sample was 98% white (*n* = 341). Most respondents 71.6% (*n* = 250) were married or in a civil partnership. Educational attainment ranged from 15.1% (*n* = 53) having no educational attainment and 32% (*n* = 110) having a first degree. Most had retired 51.2% (*n* = 183) (Table 1).

#### 3.1.1 What are the social network dimensions of cancer survivors?

The 349 participants identified 2,077 social network members (Table 4). Most frequently identified members were friends (26.1%; *n* = 546), health professionals (16.0%; *n* = 332) and children (15.5%; *n* = 322). Spouses were frequently listed (13.0%; *n* = 271), reflecting that most of the 349 participants were married. There were 1.7% (*n* = 36) pets, indicating 10% of participants listed an animal. The overall mean number of members listed in a participant's social network (network size) was 6 (SD 4.7). The overall diversity mean (different network member types, e.g. family and neighbours) was 3.6 (SD 1.8). The most commonly attended social groups (attended at least monthly) were social clubs and hobby groups (45.0%), sports and exercise groups (37.5%) and participation in volunteering groups (21.8%); 12% of participants in the study also attended religious groups or places of worship. Overall, 84.2% of participants were satisfied with their current social opportunities (Table 2).

**TABLE 2:** Cancer survivors social network membership

Relationship	Frequency	%
Partner	271	12.9
Children	322	15.4
Close family	211	10.1
Extended family	161	7.7
Friends	546	26.1
Work colleagues	89	4.3
Paid domestic support	6	0.3
Neighbours	61	2.9
Health professionals	332	15.9
Recreation group groups	42	2.0
Pets	36	1.7
Total	2077	99.2
Missing	16	0.8

#### 3.1.2 What is the self‐management work social network members contribute to?

**FIGURE 3:**
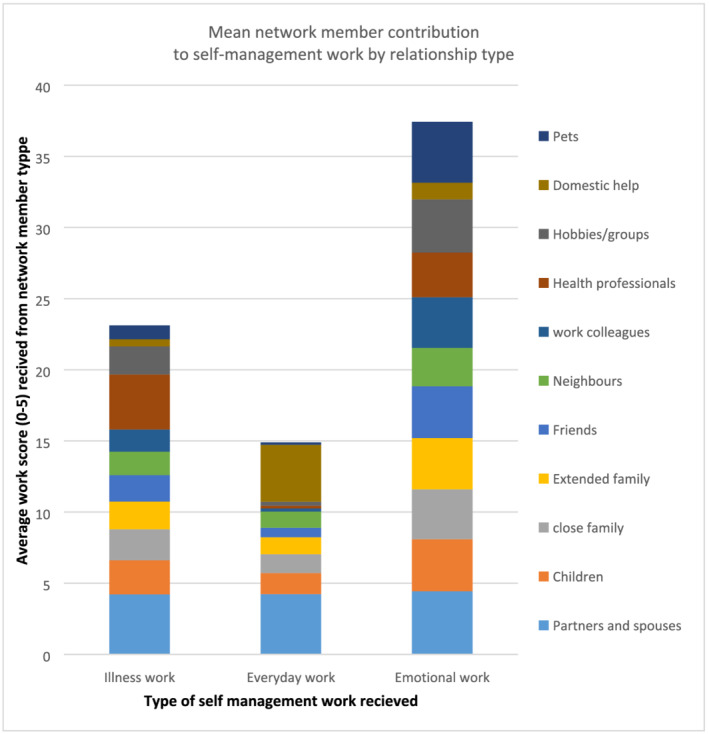
Mean network member contribution score (out of five) to self‐management by relationship type

Across the three self‐management work domains spouses contributed most to illness work (Mean [M] = 4.2), everyday work (M = 4.2) and emotional work (M = 4.4) (Figure 3). The lowest scores overall were seen in the day‐to‐day work domain. Domestic workers (providing childcare or cleaning) scored highest (M = 4.0) after spouses but this score was only found in the breast cancer (female) sub‐group. With respect to emotional work, pets achieved the highest score (M = 4.2) after spouses, followed by hobbies and groups (M = 3.7). Within illness work, health professionals contributed the largest amount of work (M = 3.8) after spouses, followed by children (M = 2.3) (Figure 3).

#### 3.1.3 What personal, social network and self‐management dimensions influence HRQoL?

Participant characteristics explained 16.4% of the variation in HRQoL (according to the adjusted *R* ^*2*^) *F*(12, 271) = 5.637, *p* < 0.001, adj *R* ^*2*^ = 0.164. There was evidence supporting non‐zero effects for five of the 12 variables: age; working status; time spent self‐managing; self‐management skills and self‐management monitoring. Social network engagement explained 16.4% of the variation in HRQoL (according to the adjusted *R* ^*2*^) *F*(18, 303) = 4.494, *p* < 0.001, adj. *R* ^*2*^ = 0.164. There was evidence supporting non‐zero effects for: satisfaction with social opportunities, weekly, monthly and less often sport/exercise participation. Network contribution to self‐management characteristics did not explain variation with HRQoL, *F*(5, 314) = 1.785, *p* > 0.05 (0.116), adj. *R* ^*2*^ = 0.012. There was evidence supporting non‐zero effects for one variable, emotional work. Social distress characteristics explained 60.2% of the variation in HRQoL (according to the adjusted *R* ^*2*^) *F*(2, 331) = 253.287,

*p* < 0.000, adj. *R* ^*2*^ = 0.602. There was evidence supporting non‐zero effects for: illness perception and social distress.

A final multiple regression model explained 63.1% of the variation in HRQoL (according to the adjusted *R* ^*2*^) *F*(13, 266) = 37.757, *p* < 0.0005, adj. *R* ^*2*^ = 0.631. There was evidence supporting non‐zero effects for: self‐management skills, sport participation (weekly, monthly and less often), social distress and illness perception *p* < 0.05. (Table 3).

**TABLE 3:** Phase 1 final composite multivariate model undertaken with Fact‐G

Variable	β	95% CI	*p*‐value
Age (years)	−0.117	−0.240, −0.006	0.062
Working status (working/retired)	−0.530	−3.461, 2.400	0.722
Time spent self‐managing (1 to 4)	−0.259	−1.119, 0.601	0.553
HEIQ self‐monitoring scale 4 (0 to 4)	−0.020	−0.428, 0.389	0.925
HEIQ skills scale 6 (0 to 4)	0.543	0.001, 1.084	0.049*
Satisfaction with social opportunities (1 to 5)	−0.707	−2.134, 720	0.330
Sport/exercise participation weekly	3.196	0.866, 5.527	0.007*
Monthly	5.072	0.475, 9.670	0.031*
3 monthly	3.681	−1.305,8.667	0.147
Less often	5.371	1.499, 9.964	0.008*
Contribution to emotional work by network members (0 to 5 per member)	−0.019	−0.076, 0.039	0.529
Social distress index (0 to 80)	−0.981	−1.194, −0.767	0.001*
Illness perception questionnaire (0 to 44)	−0.464	−0.559, −0.368	0.000*

* *p* value < .05.

Our findings indicate that wider engagement of cancer survivors with social activity and the subsequent receipt of network self‐management support improves HRQoL. SES was found to be statistically significant when simple linear regression was applied. This was not the case with multiple regression, suggesting that other effects correlated with SES were more important than SES alone.

Survey findings directed analysis aims of Phase 2 of the study. Interview data enabled exploration of why membership of social network groups might contribute towards lower social distress, increased self‐management and HRQoL and elicit mechanisms through which this occurred. Analysis also gave the opportunity to explore the subtle influence of SES on social networks and self‐management.

### 3.2 Phase 2 findings

A purposeful sample of 20 participants was recruited from the 220 survey participants who expressed an interest in being interviewed (Table 4). Qualitative analysis identified four themes that illuminated mechanisms through which different types of groups influenced participants' engagement in self‐management support and how SES contributed. The themes were; disrupted networks after cancer treatment, navigating formal support and building capacity, peer networks and self‐management, and linking networks to enable adaptation.

**TABLE 4:** Phase 2 participant characteristics

Participant number	Gender	Age	Socio economic status^a^	Illness type
ID1	Female	72	Low	Breast
ID2	Female	48	Low	Breast
ID3	Female	67	Low	Lymphoma
ID4	Male	72	Low	Lymphoma
ID5	Female	44	High	Breast
ID6	Male	52	Low	Lymphoma
ID7	Male	74	High	Prostate
ID8	Male	63	Low	Bowel
ID9	Female	45	High	Lymphoma
ID10	Female	64	Low	Bowel
ID11	Female	58	Low	Lymphoma
ID12	Male	74	Low	Prostate
ID13	Male	69	High	Bowel
ID14	Male	81	Low	Bowel
ID15	Male	32	High	Lymphoma
ID16	Female	82	Low	Breast
ID17	Male	70	Low	Prostate
ID18	Female	67	Low	Bowel
ID19	Female	67	High	Bowel
ID20	Male	64	Low	Prostate

a Socio‐ecconomic status was defined using postcode and Indices of Multiple Deprivation (IMD), range 1 to 10, 1 most deprived, 10 least deprived. Participants with IMD 1‐5 classified as having low SES and IMD 6‐10 as high SES.

#### 3.2.1 Disrupted networks after cancer treatment

Participants described significant reshaping of their social networks during and after treatment. The impact of network reshaping influenced participants' ability to engage with opportunities and resources to support self‐management in recovery. When cancer treatment was complete, participants were viewed by network members as returning to health and support withdrawn, or participants shielded close family and friends from their ongoing distress, concerned that they would be overburdened.

People think I'm better now and I'm not better now but everybody expects you to just be better now. That's what I find hard actually is everybody else's expectations I suppose. ID11, female, low SES

Participants with low SES and networks observed to be dominated by family members in Phase 2, described how they consciously dropped peripheral network members during treatment and did not re‐engage with them, choosing to rely only on family members. This was primarily due to the complex relationship work needed to maintain engagement across networks. Disruption to participants' social networks limited capacity to access self‐management resources available from within those networks, such as emotional and practical support. At treatment completion, participants described how their previous or newly reshaped social networks either contributed or prevented facilitation to navigate their re‐defined network identity to promote their health.

I sort of focused in and I focussed on the family because they were the ones looking after me. So when I got back home I got even smaller and it (social network) stayed basically with a small family …. I stopped socialising outside with friends or contacts I had. I chose, well I did it slowly. (ID18, female, low SES)

#### 3.2.2 Navigating formal support and building capacity

Cancer support offered by third sector networks gave participants the opportunity to engage in cancer‐related self‐management resources free of charge, at different time points during treatment and recovery, which promoted an increased sense of control and well‐being. Cancer support centres were valued because they offered illness‐related support beyond family and friends, consciously relieving carers from further support. Centres were also valued because they created a sense of equality between cancer survivors through the shared experience of cancer, which took priority over SES.

I did not think it was something I would need, but actually it (counselling at cancer centre) was the right thing …. You cannot load on people that are close to you all the time, I needed someone who could listen to my upset. ID05, female, high SES

#### 3.2.3 Peer networks and self‐management

Peer to peer weak tie networks with other cancer survivors appeared to be an acceptable and credible approach to share and engage in self‐management support during and after treatment. Peer network engagement created subtle opportunities to share knowledge and resources to support self‐management, which was more acceptable than formal peer support groups. Peer networks developed through weak tie groups, when the purpose was not focused on cancer management (e.g. work) were highly valued by participants. For some participants with low SES and family dominate networks, employment provided the only opportunity to develop such networks. The combination of a shared non‐cancer environment, engagement with weaker ties and shared illness experience appeared to have a beneficial impact on shaping participants' abilities to self‐manage the consequences of cancer.

There's a lady at our work went through a similar thing … she told me with reference to her (bowel) symptoms because we speak together, it took her three years after her operation before she felt she was fit enough, like before the op, so I mean I can see where she is coming from now. (ID08, male, low SES)

#### 3.2.4 Linking familiar and new networks to enable adaptation

Recreational, work and faith networks facilitated day‐to‐day adaptation in recovery. Established and new networks of non‐illness and non‐family members recognised the need to support adaptation to new physical and emotional circumstances after cancer treatment, enabling participants to engage in a full range of network activities. These weak tie networks made a valuable contribution to participants' ability to navigate their redefined relationships by giving a sense of personal fulfilment without burdening family and enabling successful adaptation to a new normal in the wider social environment.

My friend, who went to the same exercise class with me before I was ill suggested that exercise teacher might help me at home. I did not know she could do that. So the exercise teacher sees me 1:1, she has kept a slot for me, I'm very lucky. ID09, female, high SES

## 4 DISCUSSION

### 4.1 Contribution of social networks to self‐management support

Our findings indicate that weak tie network groups appear to have a positive impact on self‐management and HRQoL through engagement in a range of opportunities, resources and experiences, suggesting such networks could have a valuable and previously overlooked role in supporting cancer survivors' to self‐manage long‐term health needs.

Close family members are frequently turned to in times of acute need (Perry & Pescosolido, 2015) and our findings concur but also identified for the first time the mechanisms of how weak tie networks, such as recreational groups, informal peer networks and community cancer support services make valuable contributions to self‐management and lowering social distress after treatment. The value of network groups, particularly peer support has been recognised (Dunn et al., 2003). Our paper contextualises the contribution of peer support to self‐management within the wider focus of a network approach, while offering some insights as to why support groups may not work for all and the importance of other weak tie networks.

### 4.2 Network membership, socio‐economic status and self‐management support

Our findings suggest that characteristics of network membership, such as diversity, appeared to have more influence on ability to engage with network self‐management opportunities and that lower SES alone was not prohibitive of network group engagement. Phase 2 findings revealed that participants whose networks were dominated by family members tended to have low SES. Phase 2 findings also indicated that while low SES did not appear to restrict access to direct self‐management support it did appear to be indirectly associated with preventing access to social resources, such as transport, limiting engagement. Participants with higher SES were less impacted by access to resources and benefited from additional resources, such as occupational health services.

Participants with lower SES and family dominated networks appeared to have limited opportunities to engage with wider self‐management support and resources. This could be due to limited individual and network resources (e.g. employment flexibility) and the potential burden that self‐management support could put on network members (Kroenke et al., 2013; Perry & Pescosolido, 2015; Reeves et al., 2014; Walker et al., 2018). It is possible that participants who had embedded family networks could have been satisfied with the variety of resources already provided within their network and did not feel it necessary to seek external support. The substantial emotional and identity investment people have within families can make it challenging to renegotiate these relationships and engage in new or alternative self‐management resources (Vassilev et al., 2016). This is more difficult during a time of crisis when people's networks tend to shrink even though self‐management and quality of life might benefit from access to larger and more diverse networks.

Acknowledging the contribution of wider social networks and understanding how relationships, positive or negative, within the context of such networks may shape one another, could potentially contribute to upscaling cancer survivorship care, bridging the gap between hospital self‐management support and community social networks.

#### 4.2.1 Methodological value

The study indicated the value of adopting a mixed method network approach to illuminate self‐management support of cancer survivors. Findings suggest the influence of structural components of networks, such as size and diversity have a nuanced influence on how and why cancer survivors access or do not access resources and support, which cannot be captured and explained by only drawing on either quantitative or qualitative methods alone. Our findings also indicate that HRQoL may be too crude an outcome measure to identify the influence of social networks on cancer survivors' social distress and ability to self‐manage. Utilising a well‐being outcome measure in conjunction with HRQoL would give the opportunity to measure a variety of positive assets in functioning.

Findings also indicate data collection methods and measures used in the quantitative phase of the study tended to underestimate the involvement and role of weak ties in social networks and self‐management support, which could reflect the value cancer survivors' put on relationships with stronger ties. The value of adopting a mixed method approach was further demonstrated as the qualitative analysis demonstrated the key role weak ties play in self‐management support and quality of life is largely invisible and their value is precisely due to these links being understated by participants (Rogers et al., 2014). Developing and using measures of network engagement and support capable of capturing the role of weak ties and relationship work is likely to lead to a better understanding of the needs and experiences of cancer survivors.

### 4.3 Limitations

We recognise that a more robust sequential approach could have been adopted whereby results of the survey directly informed the interview schedule, and not just the analysis. Parallel data collection limited the strengths gained from adopting a mixed method design and could have impacted study findings. Participants were predominantly white (2% self‐identified as BME), educated, middle income and did not reflect greater ethnic diversity or a broader SES population.

The HRQoL measure Fact‐G (Cella et al., 1993) has been criticised as not reflecting the broader concerns of cancer survivors who have completed treatment (Yost et al., 2013). Future studies could consider more cancer survivor specific HRQoL measures such as the Quality of life in Adult Cancer Survivors (Avis et al., 2005). These limitations suggest the findings need to be interpreted with caution in terms of implications for the wider population.

## 5 CONCLUSIONS AND CLINICAL IMPLICATIONS

This study set out to explore if the dimensions of cancer survivors' personal social networks shaped self‐management support and their relationship with HRQoL. Taking a mixed methods approach and drawing on social network theories, findings suggest that engagement in community groups and interactions with weak tie social network members can make a substantial contribution to self‐management and improve HRQoL. This study identified the previously under‐recognised contribution of social networks, especially of weak ties, in the context of self‐management support for cancer survivors, and has implications for expansion of future health service delivery and improved health. Findings also illuminated some of the mechanisms through which SES and network structure could shape self‐management support. Future research should consider incorporating the broader construct of well‐being as well as HRQoL.

Findings suggest healthcare professionals need to recognise the wider contribution of social networks to facilitate self‐management support of cancer survivors. Healthcare staff can contribute to health and well‐being beyond clinical care by advocating survivors' engagement in non‐clinical, community cancer services and non‐cancer social interaction. Social networks are an untapped self‐management resource for cancer survivors and have significant potential to improve health and well‐being.

## CONFLICT OF INTERESTS

Gilly Howard‐Jones, Clinical Doctoral Research Fellow, NIHR CDRF‐2013‐04‐029, is funded by Health Education England (HEE)/National Institute for Health Research (NIHR) for this research project. Professor Richardson is a National Institute for Health Research (NIHR) Senior Investigator. The views expressed in this article are those of the author(s) and not necessarily those of the NHS, the NIHR or the Department of Health. The authors have no conflict of interests to declare.

## Data Availability

The data that support the findings of this study are available from the corresponding author upon reasonable request.

## References

[ecc13578-bib-0001] Avis, N. E. , Smith, K. W. , McGraw, S. , Smith, R. G. , Petronis, V. M. , & Carver, C. S. (2005). Assessing Quality of Life in Adult Cancer Survivors (QLACS). Quality of Life Research, 14(4), 1007–1023. 10.1007/s11136-004-2147-2 16041897

[ecc13578-bib-0002] Balfe, M. , Keohane, K. , O'Brien, K. , & Sharp, L. (2017). Social networks, social support and social negativity: A qualitative study of head and neck cancer caregivers' experiences. European Journal of Cancer Care, 26(6), e12619. 10.1111/ecc.12619 28004448

[ecc13578-bib-0003] Batehup, L. , Porter, K. , Gage, H. , Williams, P. , Simmonds, P. , Lowson, E. , Dodson, L. , Davies, N. J. , Wagland, R. , Winter, J. D. , Richardson, A. , Turner, A. , & Corner, J. L. (2017). Follow‐up after curative treatment for colorectal cancer: Longitudinal evaluation of patient initiated follow‐up in the first 12 months. Support Care Cancer, 25(7), 2063–2073. 10.1007/s00520-017-3595-x 28197848 PMC5445145

[ecc13578-bib-0004] Bazeley, P. (2018). Integrating analyses in mixed methods research. SAGE. 10.4135/9781526417190

[ecc13578-bib-0005] Beasley, J. M. , Newcomb, P. A. , Trentham‐Dietz, A. , Hampton, J. M. , Ceballos, R. M. , Titus‐Ernstoff, L. , Egan, K. M. , & Holmes, M. D. (2010). Social networks and survival after breast cancer diagnosis. Journal of Cancer Survivorship: Research and Practice, 4(4), 372–380. 10.1007/s11764-010-0139-5 20652435 PMC2978785

[ecc13578-bib-0006] Berkman, L. , & Syme, L. (1979). Social networks, host resistance and mortality; a nine year follow up study of Alameda County residents. American Journal of Epidemiology, 109(2), 186–204. 10.1093/oxfordjournals.aje.a112674 425958

[ecc13578-bib-0007] Broadbent, E. , Petrie, K. J. , Main, J. , & Weinman, J. (2006). The brief illness perception questionnaire. Journal of Psychosomatic Research, 60(6), 631–637. 10.1016/j.jpsychores.2005.10.020 16731240

[ecc13578-bib-0008] Bryman, A. (2016). Social research methods (5th ed.). Oxford University Press.

[ecc13578-bib-0009] Burns, N. , & Grove, S. (2003). Understanding nursing research (3rd ed., Vol. 7). Saunders.

[ecc13578-bib-0010] Cella, D. F. , Tulsky, D. S. , Gray, G. , Sarafian, B. , Linn, E. , Bonomi, A. , Silberman, M. , Yellen, S. B. , Winicour, P. , & Brannon, J. (1993). The functional assessment of cancer therapy scale: Development and validation of the general measure. Journal of Clinical Oncology, 11(3), 570–579. 10.1200/JCO.1993.11.3.570 8445433

[ecc13578-bib-0011] Cheng, H. , Sit, J. W. H. , Chan, C. W. H. , So, W. K. W. , Choi, K. C. , & Cheng, K. K. F. (2013). Social support and quality of life among Chinese breast cancer survivors: Findings from a mixed methods study. European Journal of Oncology Nursing, 17(6), 788–796. 10.1016/j.ejon.2013.03.007 23587632

[ecc13578-bib-0012] Christakis, N. A. , & Fowler, J. H. (2007). The spread of obesity in a large social network over 32 years. New England Journal of Medicine, 357(4), 370–379. 10.1056/NEJMsa066082 17652652

[ecc13578-bib-0013] Crookes, D. , Shelton, R. , Tehranifar, P. , Aycinena, C. , Gaffney, A. , Koch, P. , Contento, I. , Greenlee, H. , Crookes, D. M. , Shelton, R. C. , Gaffney, A. O. , & Contento, I. R. (2016). Social networks and social support for healthy eating among Latina breast cancer survivors: Implications for social and behavioral interventions. Journal of Cancer Survivorship, 10(2), 291–301, 11p. 10.1007/s11764-015-0475-6 26202538 PMC4724562

[ecc13578-bib-0014] Dunn, J. , Green, A. , Ralph, N. , Newton, R. U. , Kneebone, A. , Frydenberg, M. , & Chambers, S. K. (2021). Prostate cancer survivorship essentials framework: Guidelines for practitioners. BJU International, 128(S3), 18–29. 10.1111/bju.15159 PMC9291032 32627306

[ecc13578-bib-0015] Dunn, J. , Steginga, S. K. , Rosoman, N. , & Millichap, D. (2003). A review of peer support in the context of cancer. Journal of Psychosocial Oncology, 21(2), 55–67. 10.1300/J077v21n02_04

[ecc13578-bib-0016] Fenlon, D. R. , Khambhaita, P. , & Hunter, M. S. (2015). Helping patients to help themselves after breast Cancer treatment. Clinical Oncology, 27(11), 640–646. 10.1016/j.clon.2015.05.002 26047887

[ecc13578-bib-0018] Gale, N. , Heath, G. , Cameron, E. , Rashid, S. , & Redwood, S. (2013). Using the framework method for the analysis of qualitative data in multi‐disciplinary health research. BMC Medical Research Methodology, 13, 117. 10.1186/1471-2288-13-117 24047204 PMC3848812

[ecc13578-bib-0019] Gov.uk . (2015) 2015 English indices of multiple deprivation map. Gov.Uk. Available from: http://dclgapps.communities.gov.uk/imd/idmap.html]

[ecc13578-bib-0020] Granovetter, M. (1973). The strength of weak ties. American Journal of Sociology, 78(6), 1360–1380. 10.1086/225469

[ecc13578-bib-0021] Hamilton, J. B. , Moore, C. E. , Powe, B. D. , Agarwal, M. , & Martin, P. (2010). Perceptions of support among older African American cancer survivors. Oncology Nursing Forum, 37(4), 484–493. 10.1188/10.ONF.484-493 20591808 PMC2948788

[ecc13578-bib-0022] Henshall, C. L. , Greenfield, S. M. , & Gale, N. K. (2018). Typologies for restructuring relationships in cancer survivorship: Temporal changes in social support and engagement with self‐management practices. Cancer Nursing, 41(6), E32–E40. 10.1097/NCC.0000000000000538 28953505

[ecc13578-bib-0023] House, J. S. , Landis, K. R. , & Umberson, D. (1988). Social relationships and health. Science, 241(4865), 540–545. 10.1126/science.3399889 3399889

[ecc13578-bib-0024] Hughes, S. , Jaremka, L. M. , Alfano, C. M. , Glaser, R. , Povoski, S. P. , Lipari, A. M. , Agnese, D. M. , Farrar, W. B. , Yee, L. D. , Carson, W. E. , Malarkey, W. B. , & Kiecolt‐Glaser, J. K. (2014). Social support predicts inflammation, pain, and depressive symptoms: Longitudinal relationships among breast cancer survivors. Psychoneuroendocrinology, 42, 38–44. 10.1016/j.psyneuen.2013.12.016 24636499 PMC3970938

[ecc13578-bib-0025] Jones, E. C. , & Storksdieck, M. (2019). Recent research on the social network concept and cancer. Current Opinion in Supportive and Palliative Care, 13(3), 225–237. 10.1097/SPC.0000000000000442 31246595

[ecc13578-bib-0026] Juárez‐Ramírez, C. , Théodore, F. L. , Villalobos, A. , Jiménez‐Corona, A. , Lerin, S. , Nigenda, G. , & Lewis, S. (2015). Social support of patients with type 2 diabetes in marginalized contexts in Mexico and its relation to compliance with treatment: A sociocultural approach. PLoS ONE, 10(11), e0141766. 10.1371/journal.pone.0141766 26545122 PMC4636160

[ecc13578-bib-0027] Kim, B. H. , Wallington, S. F. , Makambi, K. H. , & Adams‐Campbell, L. L. (2015). Social networks and physical activity behaviors among cancer survivors: Data from the 2005 health information National Trends Survey. Journal of Health Communication, 20(6), 656–662. 10.1080/10810730.2015.1018576 25978562 PMC4507504

[ecc13578-bib-0028] Kim, S. H. , Park, S. , Kim, S. J. , Hur, M. H. , Lee, B. G. , & Han, M. S. (2020). Self‐management needs of breast cancer survivors after treatment: Results from a focus group interview. Cancer Nursing, 43(1), 78–85. 10.1097/NCC.0000000000000641 30148729

[ecc13578-bib-0029] Klein, P. (1999). The handbook of psychological testing (2nd ed.). Routledge.

[ecc13578-bib-0030] Kroenke, C. H. , Kubzansky, L. D. , Schernhammer, E. S. , Holmes, M. D. , & Kawachi, I. (2006). Social networks, social support, and survival after breast Cancer diagnosis. Journal of Clinical Oncology, 24(7), 1105–1111. 10.1200/JCO.2005.04.2846 16505430

[ecc13578-bib-0031] Kroenke, C. H. , Kwan, M. L. , Neugut, A. I. , Ergas, I. J. , Wright, J. D. , Caan, B. J. , Hershman, D. , & Kushi, L. H. (2013). Social networks, social support mechanisms, and quality of life after breast cancer diagnosis. Breast Cancer Research and Treatment, 139(2), 515–527. 10.1007/s10549-013-2477-2 23657404 PMC3906043

[ecc13578-bib-0032] Kroenke, C. H. , Michael, Y. L. , Poole, E. M. , Kwan, M. L. , Nechuta, S. , Leas, E. , Caan, B. J. , Pierce, J. , Shu, X.‐O. , Zheng, Y. , & Chen, W. Y. (2017). Postdiagnosis social networks and breast cancer mortality in the after breast Cancer pooling project. Cancer, 123(7), 1228–1237. 10.1002/cncr.30440 27943274 PMC5360517

[ecc13578-bib-0033] Kvale, S. (2007). ng interviews. Sage. 10.4135/9781849208963

[ecc13578-bib-0034] Lim, B. , & Zebrack, B. (2006). Social networks and quality of life for long‐term survivors of leukemia and lymphoma. Supportive Care in Cancer: Official Journal of the Multinational Association of Supportive Care in Cancer, 14(2), 185–192. 16007454 10.1007/s00520-005-0856-x

[ecc13578-bib-0035] Lincoln, Y. S. , & Guba, E G . (1985). Naturalistic inquiry. SAGE 9 4 438 439 10.1016/0147-1767(85)90062-8

[ecc13578-bib-0036] Lindstrom, M. , & Rosvall, M. (2019). Two theoretical strands of social capital, and total, cardiovascular, cancer and other mortality: A population‐based cohort study. Social Science & Medicine, 7, 100337. 10.1016/j.ssmph.2018.100337 PMC6302214 30623011

[ecc13578-bib-0037] Maddams, J. , Utley, M. , & Moller, H. (2012). Projections of cancer prevalence in the United Kingdom, 2010‐2040. British Journal of Cancer, 107(7), 1195–1202. 10.1038/bjc.2012.366 22892390 PMC3461160

[ecc13578-bib-0038] Maunsell, E. , Lauzier, S. , Brunet, J. , Pelletier, S. , Osborne, R. H. , & Campbell, H. S. (2014). Health‐related empowerment in cancer: Validity of scales from the health education impact questionnaire. Cancer, 120(20), 3228–3236. 10.1002/cncr.28847 24988944

[ecc13578-bib-0039] O'Brien, B. C. , Harris, I. B. , Beckman, T. J. , Reed, D. A. , & Cook, D. A. (2014). Standards for reporting qualitative research: A synthesis of recommendations. Academic Medicine, Sep;89(9), 1245, 24979285–1251. 10.1097/ACM.0000000000000388 24979285

[ecc13578-bib-0040] O'Cathain, A. , Murphy, E. , & Nicholl, J. (2007). Integration and publications as indicators of “yield.” From mixed methods studies. Journal of Mixed Methods Research, 1(2), 147–163. 10.1177/1558689806299094

[ecc13578-bib-0041] O'Cathain, A. , Murphy, E. , & Nicholl, J. (2008). The quality of mixed methods studies in health services research. Journal of Health Services Research & Policy, 13(2), 92–98. 10.1258/jhsrp.2007.007074 18416914

[ecc13578-bib-0042] Osborne, R. H. , Elsworth, G. R. , & Whitfield, K . (2007). The Health Education Impact Questionnaire (HEIQ): An outcomes and evaluation measure for patient education and self‐management interventions for people with chronic conditions. Patient Education and Counseling 66(2): 192–201 10p 10.1016/j.pec.2006.12.002 17320338

[ecc13578-bib-0043] Paterson, C. , Robertson, A. , & Nabi, G. (2015). Exploring prostate cancer survivors' self‐management behaviours and examining the mechanism effect that links coping and social support to health related quality of life, anxiety and depression: A prospective longitudinal study. European Journal of Oncology Nursing, 19, 120–128. 10.1016/j.ejon.2014.10.008 25497067

[ecc13578-bib-0044] Perry, B. L. , & Pescosolido, B. A. (2015). Social network activation: The role of health discussion partners in recovery from mental illness. Social Science & Medicine, 125, 116–128. 10.1016/j.socscimed.2013.12.033 24525260 PMC4110193

[ecc13578-bib-0045] Quin Patton, M . (2002). Qualitative research and evaluation methods (3rd Edition). Sage

[ecc13578-bib-0046] Reeves, D. , Blickem, C. , Vassilev, I. , Brooks, H. , Kennedy, A. , Richardson, G. , & Rogers, A. (2014). The contribution of social networks to the health and self‐management of patients with long‐term conditions: A longitudinal study. PLoS ONE, 9(6), e98340. 10.1371/journal.pone.0098340 24887107 PMC4041782

[ecc13578-bib-0047] Ritchie, J. , & Lewis, J. (2014). Qualitative research practice. Sage.

[ecc13578-bib-0048] Rogers, A. , Brooks, H. , Vassilev, I. , Kennedy, A. , Blickem, C. , & Reeves, D. (2014). Why less may be more: A mixed methods study of the work and relatedness of ‘weak ties’ in supporting long‐term condition self‐management. Implementation Science, 9(19). 10.1186/1748-5908-9-19 PMC3932842 24524253

[ecc13578-bib-0049] Sarma, E. A. , Kawachi, I. , Poole, E. M. , Tworoger, S. S. , Giovannucci, E. L. , Fuchs, C. S. , & Bao, Y. (2018). Social integration and survival after diagnosis of colorectal cancer. Cancer, 124(4), 833–840. 10.1002/cncr.31117 29160897 PMC5800969

[ecc13578-bib-0050] Soares, A. , Biasoli, I. , Scheliga, A. , Baptista, R. L. , Brabo, E. P. , Morais, J. C. , Werneck, G. L. , & Spector, N. (2013). Association of social network and social support with health‐related quality of life and fatigue in long‐term survivors of Hodgkin lymphoma. Supportive Care in Cancer: Official Journal of the Multinational Association of Supportive Care in Cancer, 21(8), 2153–2159. 10.1007/s00520-013-1775-x 23475196

[ecc13578-bib-0061] Spradley, J. (1979). Making an ethnographic record. In The Ethnographic Interview (pp. 69–76). Holt, Rinehart and Winston.

[ecc13578-bib-0051] Tashakkori, A. , & Teddlie, C. (2010). Overview of contemporary issues in mixed methods research. In Handbook of mixed methods research for the social & behavioral sciences (pp. 1–41). SAGE.

[ecc13578-bib-1051] Teddlie, C. , Tashakkori, A. , & Teddlie, C . (2010). Overview of contemporary issues in mixed methods research. In Handbook of mixed methods research for the social & behavioral sciences (pp. 1–41). SAGE.

[ecc13578-bib-0052] Teddlie, C. , & Tashakkori, A. (2009). Foundations of mixed methods research. Sage.

[ecc13578-bib-0053] Vassilev, I. , Rogers, A. , Blickem, C. , Brooks, H. , Kapadia, D. , Kennedy, A. , Sanders, C. , Kirk, S. , & Reeves, D. (2013). Social networks, the ‘work’ and work force of chronic illness self‐management: A survey analysis of personal communities. PLoS ONE, 8(4), e59723. 10.1371/journal.pone.0059723 23565162 PMC3615067

[ecc13578-bib-0054] Vassilev, I. , Rogers, A. , Kennedy, A. , Oatley, C. , & James, E. (2019). Identifying the processes of change and engagement from using a social network intervention for people with long‐term conditions. A qualitative study. Health Expectations, 22(2), 173–182. 10.1111/hex.12839 30318769 PMC6433331

[ecc13578-bib-0055] Vassilev, I. , Rogers, A. , Kennedy, A. , Wensing, M. , Koetsenruijter, J. , Orlando, R. , Portillo, M. C. , & Culliford, D. (2016). Social network type and long‐term condition management support: A cross‐sectional study in six European countries. PLoS ONE, 11(8), e0161027. 10.1371/journal.pone.0161027 27536988 PMC4990169

[ecc13578-bib-0056] Walker, S. , Kennedy, A. , Vassilev, I. , & Rogers, A. (2018). How do people with long‐term mental health problems negotiate relationships with network members at times of crisis? Health Expectations: An International Journal of Public Participation in Health Care & Health Policy, 21(1), 336–346. 10.1111/hex.12620 29024284 PMC5750694

[ecc13578-bib-0057] Waxler‐Morrison, N. , Hislop, T. G. , Mears, B. , & Kan, L. (1991). Effects of social relationships on survival for women with breast cancer: A prospective study. Social Science & Medicine, 33(2), 177–183. 10.1016/0277-9536(91)90178-F 1887281

[ecc13578-bib-0058] Wright, P. , Smith, A. B. , Keding, A. , & Velikova, G. (2011). The social difficulties inventory (SDI): Development of subscales and scoring guidance for staff. Psycho‐Oncology, 20(1), 36–43. 10.1002/pon.1705 20186841

[ecc13578-bib-0059] Yost, K. , Thompson, C. A. , Eton, D. T. , Allmer, C. , Ehlers, S. L. , Habermann, T. M. , Shanafelt, T. D. , Maurer, M. J. , Slager, S. L. , Link, B. K. , & Cerhan, J. R. (2013). The functional assessment of cancer therapy‐general (FACT‐G) is valid for monitoring quality of life in patients with non‐Hodgkin lymphoma. Leukemia and Lymphoma, 54(2), 290–297. 10.3109/10428194.2012.711830 22799432 PMC3665161

